# A comprehensive role evaluation and mechanism exploration of POGLUT2 in pan-cancer

**DOI:** 10.3389/fonc.2022.962540

**Published:** 2022-09-08

**Authors:** Xianyun Xu, Guangming Xie, Mingfeng Xie, Qian Liu

**Affiliations:** ^1^ Department of Clinical Laboratory, Affiliated Hospital of Jiangxi University of Chinese Medicine, Nanchang, China; ^2^ School of Medicine, Tongji University, Shanghai, China; ^3^ Department of Pediatric Surgery, the First Affiliate Hospital of Gannan Medical University, Ganzhou, China; ^4^ Jiangxi Provincial Clinical Research Center for Vascular Anomalies, Gannan Medical University, Ganzhou, China; ^5^ Jiangxi Province Key Research Laboratory of Chinese Medicine for the Prevention and Treatment of Hemangioma, Jiangxi University of Chinese Medicine, Nanchang, China

**Keywords:** POGLUT2, pan cancer, breast cancer, bioinformatic analysis, immune infiltration

## Abstract

**Objective:**

To evaluate the role of POGLUT2 in pan-cancer through bioinformatics analysis and experimental verification.

**Methods:**

Expression, gene mutation and amplification, methylation, and copy number alteration (CNA) of POGLUT2 were evaluated using The Cancer Genome Atlas (TCGA), Cancer Cell Line Encyclopedia (CCLE), and Genotype-Tissue Expression (GTEx) databases. Moreover, POGLUT2 on survival and disease progression in pan-cancer was performed using TCGA data. Immune infiltration and tumor microenvironment evaluations were assessed by ImmuneScore, ImmuCellAI, and TIMER databases. POGLUT2 correlated drug resistance analysis was performed using the GDSC2 database. Furthermore, POGLUT2 knockdown of breast cancer cells was established, followed by *in vitro* biological function assays and *in vivo* tumor growth study. The mechanisms of POGLUT2 in breast cancer were briefly evaluated *via* its connection with Notch signaling.

**Results:**

Increased levels of POGLUT2 were found in multiple types of cancer tissues and cell lines. Moreover, increased gene mutation and amplification, methylation, and CNA of POGLUT2 were found in several types of cancers. POGLUT2 was mainly expressed in stromal cells as verified by StromalScore, ESTIMATEScore, ImmuneScore, and Tumor purity, and POGLUT2 was positively correlated with cancer-associated fibroblasts, macrophages, monocytes, and neutrophils in the tumor microenvironment. *In vitro* and *in vivo* results showed that POGLUT2 knockdown could delay tumor growth and progression. Notch signaling components were related to the function of POGLUT2.

**Conclusions:**

Increased levels of POGLUT2 could result in the dysregulated immune cell infiltration and tumor microenvironment and showed a significant regulatory effect on the progression of breast cancer through Notch-related signaling.

## 1 Introduction

Cancer and its related mortality are one of the most serious public health problems worldwide (1). With the advancement of science and technology, novel diagnostic therapies (such as chimeric antigen receptor T cell (CAR-T) and immune checkpoint monoclonal antibody treatment (2)) tools are emerging and bringing the hope to cure the disease; however, the objective to achieve an idea survival time has not been fulfilled (3). Moreover, increased financial burden (4) and life quality improvement requests remain the main troubles for the patients and their families (5). Therefore, it is necessary to study the molecular mechanisms involved in cancer in detail (6), thereby helping to solve the above problems.

Protein *O*-glucosyltransferase 2 (POGLUT2), a gene located on chromosome 5, is a protein product located at the lumen of the endoplasmic reticulum. As a member of the endoplasmic reticulum protein family, POGLUT2 functions to prevent the secretion of all endoplasmic reticulum resident proteins *via* receptor–ligand interaction with a protein in the Golgi apparatus by a Lys-Asp-Glu-Leu or KDEL motif located at its C-terminus (7, 8). On the biochemistry function, POGLUT2 can catalyze to transfer the glucose from UDP-glucose to a serine residue and specifically target extracellular epidermal growth factor (EGF) repeats of proteins such as Notch signaling members (NOTCH1 and NOTCH3) (9, 10), thereby affecting Notch signaling pathway (11). However, its role in cancer has not been determined yet.

In the present study, we aimed to evaluate the role of POGLUT2 in pan-cancer through bioinformatics analysis and experimental verification.

## 2 Materials and methods

### 2.1 Collection and processing of data

#### 2.1.1 Differential expression analysis

The Cancer Genome Atlas (TCGA) pan-cancer, Cancer Cell Line Encyclopedia (CCLE), the Genotype-Tissue Expression (GTEx), and corresponding clinical data of the patients were downloaded from the University of California Santa Cruz (UCSC) Xena browser (https://xenabrowser.net/datapages/). Differential expression analysis of the genes in pan-caner tissue and normal tissues was performed using the combined data from TCGA and GTEx and processed with the ggplot2 package in R, whereas the mean expression of POGLUT2 in tumor tissue from TCGA, normal tissue from GTEx, and cell lines from CCLE were processed using the ggradar package in R. The protein expression of POGLUT2 was evaluated using the data from the UALCAN database (12) (http://ualcan.path.uab.edu).

#### 2.1.2 Gene mutation and amplification, methylation, and copy number alteration analysis

The data on gene mutation, amplification, and methylation was obtained from the cBioportal database (https://www.cbioportal.org/) (13). The association analysis was calculated using Pearson’s correlation method and plotted using the ggplot2 package in R.

#### 2.1.3 Enrichment analysis

Gene Set Variation Analysis (GSVA) (14) was carried out using the GSVA package, and the results were plotted using the ggplot2 and ggpubr packages in R. Gene Set Enrichment Analysis (GSEA) (15) was performed using the clusterpofiler package, and corresponding plots were fulfilled with the pheatmap package in R.

### 2.2 Kaplan–Meier curve construction for survival and disease progression analyses

The effects of POGLUT2 on four survival and disease progression-related parameters, including overall survival (OS), progression-free interval (PFI), disease-free survival (DFI), and disease-specific survival (DSS), were calculated using the expression data and clinical data obtained above and plotted using the survminer and survival packages in R. Moreover, optimal cutoff values were calculated according to Youden’s index and using the survminer and survival packages in R.

### 2.3 Association of POGLUT2 with immune infiltration and tumor microenvironment

Stromalcore, ImmuneScore, and ESTIMAScore (sum of Stromalcore and ImmuneScore) (16) were calculated using the ESTIMATE package in R. The Tumor Immune Estimation Resource (TIMER) database (17) was employed for the characterization of the cells and pathways involved in the effects of POGLUT2. Immune infiltration analysis was carried out using the CIBERSORT database (https://cibersort.stanford.edu) (18). Immune Cell Abundance Identifier (19) (ImmuCellAI; http://bioinfo.life.hust.edu.cn/ImmuCellAI#!/) was also employed for immune infiltration score calculation. The plots were achieved by either the ggplot2 or circlize package in R.

### 2.4 Drug resistance analysis

Drug resistance analysis was fulfilled using data from the Genomics of Drug Sensitivity in Cancer 2 (GDSC2) database (https://www.cancerrxgene.org/) and processed using Spearman’s correlation method.

### 2.5 Cell line culture and establishment of POGLUT2 knockdown cells

Breast cancer cell lines MDA-MB-468, MDA-MB-231, MCF-7, and BT474 were purchased from American Type Culture Collection (ATCC, Gaithersburg, MD, USA). Cells were grown in Dulbecco’s modified Eagle’s medium (DMEM) or 1640 medium supplemented with 10% fetal bovine serum (FBS; Corning, CA, USA) and 1% penicillin–streptomycin solution (Beyotime, Nantong, China) and maintained at 37°C, 5% CO_2_ condition. POGLUT2 knockdown MCF-7 and MDA-MB-231 cell line establishment was achieved by GenePharma Gene Co., Ltd. (Shanghai, China) and verified through quantitative PCR and Western blotting experiment described below.

### 2.6 Immunohistochemistry staining of POGLUT2 in breast cancer tissues

Three paired breast cancer tissue and corresponding para-tumor normal tissues were obtained and processed for series alcohol fixation, paraffin embedding, and sections. Then the sections were further handled for antigen retrieval, blocking, primary and secondary antibody staining, and detection. The antibody used for POGLUT2 staining was from NOVUS (1:200, NBP1-97469, CA, USA), whereas the two-step detection kit was from ebiogo Co., Ltd. (B001, Shanghai, China).

### 2.7 *In vitro* cell proliferation, apoptosis, cell cycle, clone formation, wound healing assay, cell migration, and invasion assay

Cell proliferation was assayed using the Cell Counting Kit-8 (CCK-8) reagent from Biomiky (BL001B, China). Briefly, 10 µl of CCK-8 was added into 100 µl of cell suspension in a 96-well plate, and the assay was performed at different time points.

Cell apoptosis was assayed using the Annexin V-PI staining kit Beyotime (22837, China). Briefly, cells were seeded in a six-well plate and grown for 48 h, followed by trypsinization, cell suspension preparation, phosphate-buffered saline (PBS) wash, binding buffer suspension, and Annexin V-PI staining for 30 min before undergoing analysis by a FACS analyzer (BD FACSCanto, San Jose, CA, USA).

Cell cycle assay was also performed using the propidium iodide (PI) staining kit from Beyotime (C1052, China). Briefly, cells were seeded in a 6-cm dish and grown into 80% confluency, followed by trypsinization, cell suspension preparation, PBS wash, ethanol fixation, and PI staining for 30 min before undergoing analysis by a FACS analyzer.

Clone formation was carried out as follows: (0.4–1) × 10^3^ cells/well were seeded in six-well plates and cultured for 14 days for clone formation. Then the wells were fixed with 4% paraformaldehyde, washed with PBS, stained with crystal violet for 20 min at room temperature, and photographed.

The wound healing assay was carried out as follows: cells were seeded into a 12-well plate and grown into 95% confluency, followed by a vertical scape made by 200 µl pipette tips and 24- or 48-h incubation in 37°C, 5% CO_2_ condition before being photographed under a microscope. Cell migration ability quantification was processed *via* measurement of the width of the wound area in the images.

Transwell cell migration and invasion were assayed in a similar manner except that a Matrigel was added in the upper chamber in the setting of invasion before cell seeding. The upper chamber was seeded with 5 × 10^4^ cells/well, and the lower chamber was added with 600 µl of complete medium with 20% FBS. Then the chamber was subjected to 37°C, 5% CO_2_ condition for a specific time period before undergoing fixation, washing, staining with crystal violet, and imaging. Three random fields (×100) were selected in each upper chamber for data processing.

### 2.8 *In vivo* tumor formation assay

Control siRNA and POGLUT2 siRNA MCF-7 cancer cells (2 × 10^7^) were subcutaneously administrated into the back of the nude mice aged 6 weeks (each cell type with six mice). The tumor growth was monitored for a period of 28 days by measuring the length (L) and width (W) using the following equation: volume = (W^2^ × L)/2. Animals were maintained under a cycle of 12/12 h (light/dark) at 24°C with free access to water and food. At the end time point, all the mice were euthanized with an overdose of pentobarbital sodium, and the tumors were isolated, photographed, and weighed for data collection.

### 2.9 Quantitative PCR

RNA from the Control siRNA and POGLUT2 siRNA-treated cells or different types of breast cancer cells were isolated using TRIzol^®^ Plus RNA Purification Kit (12183-555; Invitrogen, Carlsbad, CA, USA) according to the manufacturer’s instructions. Reverse transcription and quantitative PCR were respectively carried out using PrimeScript™ RT Master Mix (RR036A; Takara, Dalian, China) and TB Green Premix Ex Taq II (RR820A; Takara). Quantification of the relative expression levels was performed using the 2^−ΔΔCT^ method. The sequences of used primers were as follows: POGLUT2 forward 5′-TGGCAGGAGCAACTCAAGTT-3′, reverse 5′-CCCTGGCGTTCTGCTGTC-3′; GAPHD forward 5′-GATGAGATTGGCCCGATG-3′; reverse 5′-GACTGAGATTGGCCCGATG-3′.

### 2.10 Western blotting

Cell proteins were prepared from the above-indicated cells using T-PER Tissue Protein Extraction Reagent containing Halt Protease and Phosphatase Inhibitor Cocktail (Thermo Fisher, Pleasanton, CA, USA). Then cell protein was measured using BCA kit from Beyotime (P0010) before being subjected to sodium dodecyl sulfate–polyacrylamide gel electrophoresis (SDS-PAGE) electrophoresis, polyvinylidene difluoride (PVDF) membrane transferring, 5% milk blocking, and primary and secondary antibody incubation. The antibody used for POGLUT2 detection was from SANTA CRUZ (1:1,000, sc-390065, Santa Cruz, CA, USA); simultaneously, the GAPDH antibody from Proteintech Co., Ltd. (Chicago, IL, USA; 10494-1-AP) was employed as the internal control. For the experiments on Notch signaling evaluation, antibodies were from Abcam (Cambridge, UK; ab234987 for CSL) and Proteintech (55114-1-AP for Notch3; 28580-1-AP for Notch2; 66890-1-Ig for Jagged1; 20687-1-AP for Notch1). Quantification of the intensity of the protein was determined using ImageJ software (NIH, MA, USA).

## 3 Results

### 3.1 Expression analysis of POGLUT2 in pan-cancer and its correlation with clinical pathology

**Figure 1:**
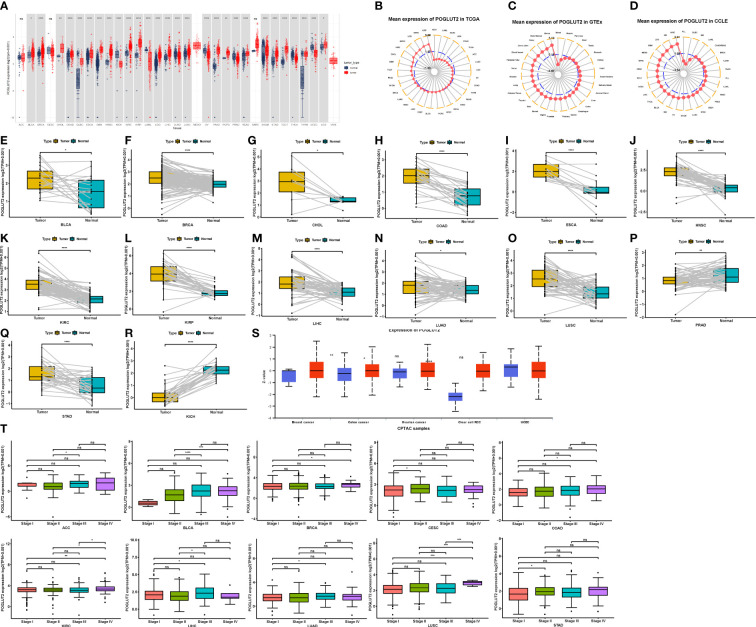
Expression pattern of POGLUT2 mRNA in different types of tumors and paired tumor and para-tumor normal tissues. **(A)** Compared to normal tissue, elevated and decreased levels of POGLUT2 mRNA were respectively found in BLCA, BRCA, CHOL, COAD, DLBC, ESCA, GBM, HNS, KIRC, KIRP, LAML, LGG, LIHC, LUAD, LUSC, OV, PAAD, PCPG, PRAD, READ, STAD, TGCT, THCA, THYM, UCS, UVM, and KICH, OV, THCA, and UCEC, whereas no difference was found in ACC, CESC, MESO, and SARC. **(B)** Mean expression of POGLUT2 mRNA in different types of tumors from TCGA. **(C)** Mean expression of POGLUT2 mRNA in different types of tumors from GTEx. **(D)** Mean expression of POGLUT2 mRNA in different types of tumors from CCLE. Elevated **(E–O)** and decreased **(P–R)** levels of POGLUT2 mRNA in paired tumor and para-tumor normal tissues. **(S)** Expression of POGLUT2 protein in different types of tumors. Elevated POGLUT2 in tumor tissue compared to normal tissue was found in breast cancer, colon cancer, and renal clear cell carcinoma, whereas no difference was found in ovarian cancer and UCEC (data are from UALCAN database). **(T)** The relationship between POGLUT2 mRNA level and pathology stages in different types of tumors. Elevated POGLUT2 mRNA level was found in tumors derived from high stages compared to lower stages. TCGA, The Cancer Genome Atlas; GTEx, Genotype-Tissue Expression; CCLE, Cancer Cell Line Encyclopedia. ns, no significant. *P < 0.05, **P < 0.01, ***P < 0.001.

Compared to normal tissue, elevated and decreased levels of POGLUT2 mRNA were found in BLCA, BRCA, CHOL, COAD, DLBC, ESCA, GBM, HNS, KIRC, KIRP, LAML, LGG, LIHC, LUAD, LUSC, OV, PAAD, PCPG, PRAD, READ, STAD, TGCT, THCA, THYM, UCS, UVM, KICH, OV, THCA, and UCEC, whereas no difference was found in ACC, CESC, MESO, and SARC (**Figure 1A**). Moreover, the elevated mean expression of POGLUT2 was confirmed in tumor tissue and cancer cell lines (**Figures 1B–D**). In the results of pair tissue comparison, elevated POGLUT2 gene levels were found in most tumor tissue compared to normal tissue except in PRAD and KICH (**Figures 1E–R**). Moreover, elevated POGLUT2 in tumor tissue compared to normal tissue was found in breast cancer, colon cancer, and renal clear cell carcinoma, whereas no difference was found in ovarian cancer and UCEC according to the data from the UALCAN database (**Figure 1S**). If the clinical data were combined, then an elevated POGLUT2 mRNA level was found in tumors derived from high stages compared to lower stages (**Figure 1T**).

### 3.2 The role of POGLUT2 in gene mutation and amplification, methylation, and copy number alteration

**Figure 2:**
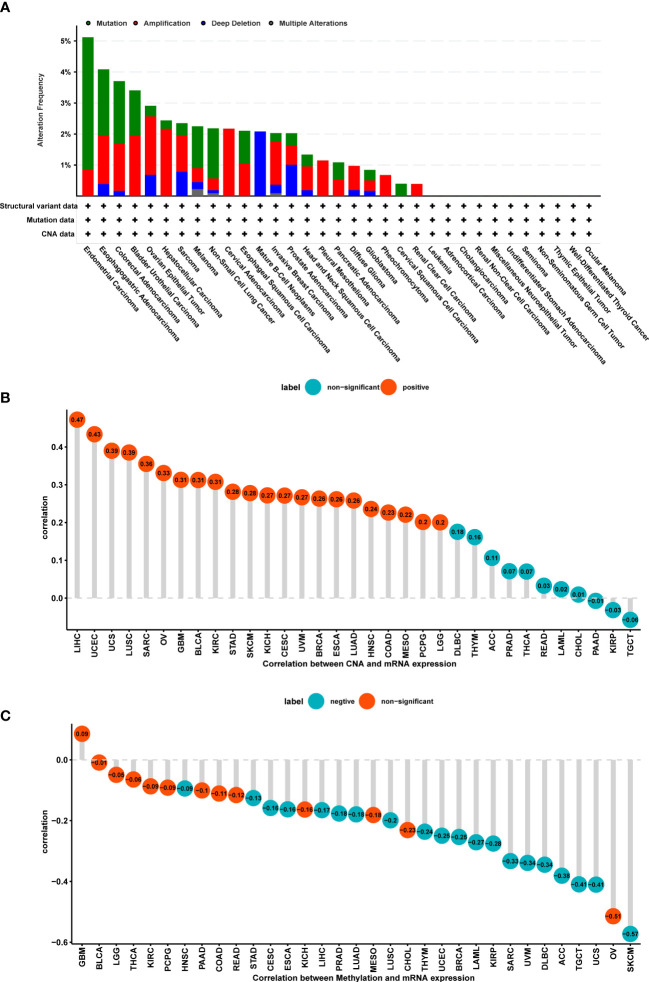
The role of POGLUT2 in gene mutation and amplification, methylation, and copy number alterations (CNAs). **(A)**. Ratio of gene mutation (green), amplification (red), deep deletion (blue), and multiple alterations (gray) in different types of tumors. **(B)** Correlation between CAN and POGLUT2 expression. Significant positive correlations were found in LIHC, UCEC, UCS, LUSC, SARC, OV, GBM, BLCA, KIRC, STAD, SKCM, KICH, CESC, UVM, BRCA, ESCA, LUAD, HNSC, COAD, MESO, and PCPG, whereas no correlation was found in LGG, DLBC, THYM, ACC, PRAD, THCA, READ, LAML, CHOL, PAAD, KIRP, and TGCT. **(C)** Correlation between methylation and POGLUT2 mRNA expression. Significant negative correlations between methylation and POGLUT2 mRNA expression were found in HNSC, STAD, CESC, ESCA, LIHC, PRAD, LUAD, LUSC, THYM, UCEC, BRCA, LAML, KIRP, SARC, UVM, DLBC, ACC, TGCT, UCS, and SKCM, but not in other types of cancers.

Considering the critical role of gene mutation and amplification, methylation, and copy number alteration (CNA) in tumor development and progression, we also plotted figures that showed the ratio variation of POGLUT2 in different types of cancers (**Figure 2A**). Moreover, the correlation parameter calculation results indicated that on CNA and POGLUT2 expression, significant positive correlations were found in LIHC, UCEC, UCS, LUSC, SARC, OV, GBM, BLCA, KIRC, STAD, SKCM, KICH, CESC, UVM, BRCA, ESCA, LUAD, HNSC, COAD, MESO, and PCPG, whereas no correlation was found in LGG, DLBC, THYM, ACC, PRAD, THCA, READ, LAML, CHOL, PAAD, KIRP, and TGCT (**Figure 2B**). On the relationship between methylation and POGLUT2 mRNA expression, significant negative correlations between methylation and POGLUT2 mRNA expression were found in HNSC, STAD, CESC, ESCA, LIHC, PRAD, LUAD, LUSC, THYM, UCEC, BRCA, LAML, KIRP, SARC, UVM, DLBC, ACC, TGCT, UCS, and SKCM but not in other types of cancers (**Figure 2C**).

### 3.3 The impact of POGLUT2 on survival and disease progression in pan-cancer

**Figure 3:**
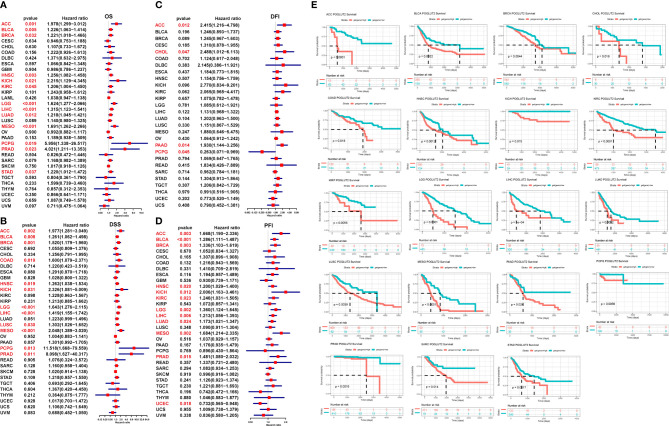
The impact of POGLUT2 on survival of pan-cancer by univariate logistic regression and receiver operating characteristic (ROC) curves. Red characters represent the data of significance. **(A)** Overall survival (OS). **(B)** Disease-specific survival (DSS). **(C)** Disease-free interval (DFI). **(D)** Progression-free interval (PFI). **(E)** ROC curve for survival in pan-cancer classified by high and low expression of POGLUT2.

We further analyze the impact of POGLUT2 on survival and disease progression in pan-cancer; the results showed that POGLUT2 could affect the OS in ACC, BLCA, BRCA, HNSC, KICH, KIRC, LGG, LIHC, LUAD, MESO, PCPG, PRAD, and STAD; DSS in ACC, BLCA, BRCA, COAD, HNSC, KICH, LGG, LIHC, LUSC, MESO, PCPG, and PRAD, but not in other types of cancers; DFI in ACC, CHOL, PAAD, and PCPG; and PFI in ACC, BLCA, BRCA, HNSC, KICH, KIRC, LGG, LIHC, LUAD, MESO, and PRAD, but not in other types of cancers (**Figures 3A–D**). Moreover, the high- and low-expression grouping also confirmed that significantly increased survival in POGLUT2 low-expression patients in most types of cancers (**Figure 3E**).

### 3.4 Enrichment analysis of POGLUT2 in breast cancer

**Figure 4:**
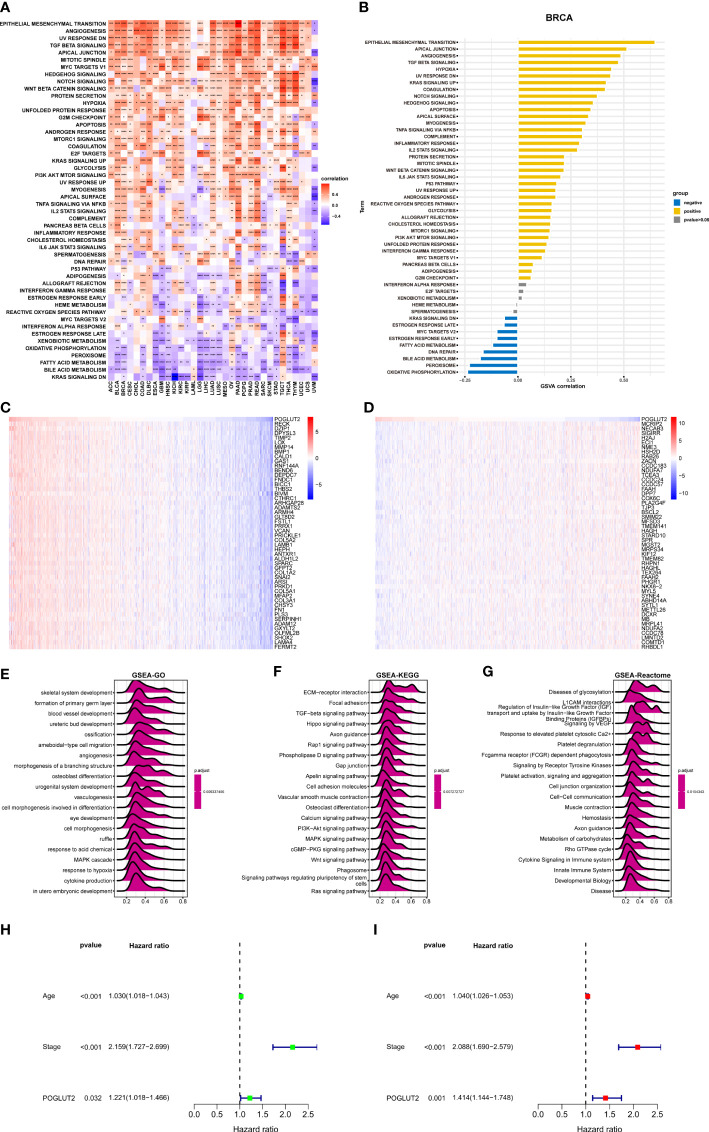
GSVA enrichment analysis. **(A)**. Top 50 POGLUT2 correlated hallmark pathways in pan-cancer. **(B)** Top 50 POGLUT2 correlated hallmark pathways in breast cancer. **(C)** Heatmap of top 50 positively correlated genes. **(D)** Heatmap of top 50 negative correlated genes. **(E)** GSEA-GO. **(F)** GSEA KEGG. **(G)** GSEA Reactome enrichment analysis using the correlation genes. Top 20 results were illustrated. **(H, I)** Univariate **(H)** and multivariate **(I)** Cox regression analyses assessing the prognostic value of POGLUT2 in breast cancer. GSVA, Gene Set Variation Analysis; GSEA, Gene Set Enrichment Analysis; KEGG, Kyoto Encyclopedia of Genes and Genomes.

We also performed GSVA and GSEA. In GSVA, epithelial–mesenchymal transition, apical junction, and angiogenesis are the top 3 upregulated pathways that correlated with POGLUT2, whereas oxidative phosphorylation, peroxisome, and bile acid metabolism are the top 3 downregulated pathways that correlated with POGLUT2 (**Figures 4A, B**) In GSEA, we listed the heatmap of the top 50 positive and negative correlated genes of POGLUT2, and the top 3 were respectively RECK, DZIP1, and DPYSL3 and MCRIP2, NECAB3, and SIGIRR (**Figures 4C, D**). Moreover, the GSEA-GO, Kyoto Encyclopedia of Genes and Genomes (KEGG), and Reactome results were also listed, and the top 3 terms were skeletal system development, formation of the primary germ layer, and blood vessel development in GO; extracellular matrix (ECM)–receptor interaction, focal adhesion, TGF-beta signaling in KEGG; and disease of glycosylation, L1CAM interaction, and regulation of insulin-like growth factor in Reactome (**Figures 4E–G**). Moreover, the univariate (**Figure 4H**) and multivariate (**Figure 4I**) Cox regression analyses demonstrated that POGLUT2 (*p* < 0.05) acted as an independent prognostic indicator of breast cancer together with age and stage (*p* < 0.001).

### 3.5 The effects of POGLUT2 on immune cell infiltration and tumor microenvironment

**Figure 5:**
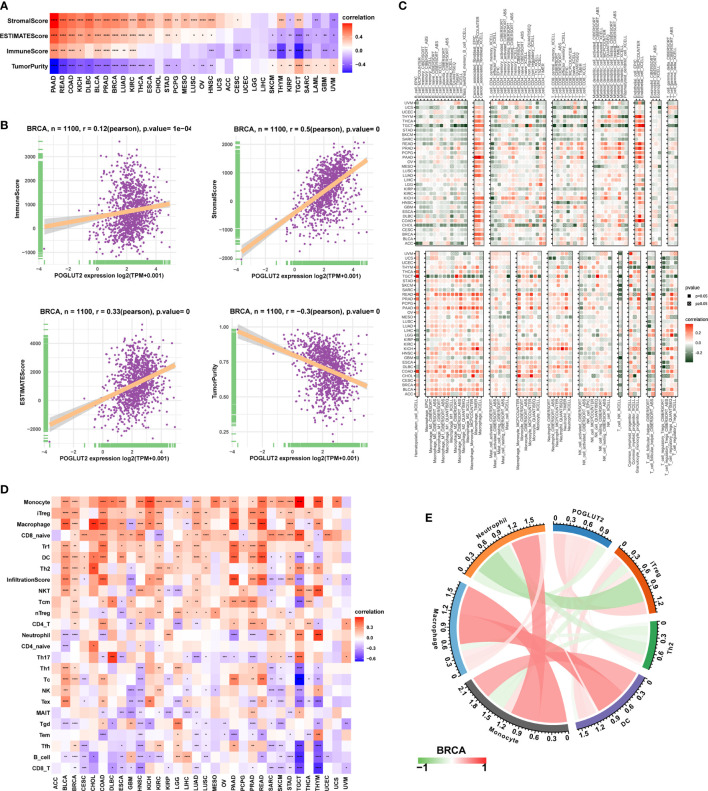
The role of POGLUT2 in tumor microenvironment. **(A)** POGLUT2 correlated Stromal Score, ESTIMATEScore, ImmuneScore, and Tumor purity in different types of cancers. **(B)** The relationship between POGLUT2 mRNA level and ImmuneScore, Stromal Score, ESTIMATEScore, and Tumor purity in BRCA dataset. **(C)** The role of POGLUT2 on immune infiltration using the data from the TIMER2 database. **(D)** The role of POGLUT2 on immune cell infiltration using data from ImmuCellAI database (http://bioinfo.life.hust.edu.cn/ImmuCellAI#!/). **(E)** The role of POGLUT2 on immune cell infiltration in the setting of breast cancer.

We also calculated the effects of POGLUT2 on immune cell infiltration. Firstly, a significant correlation could be found between POGLUT2 and StromalScore, ESTIMATEScore, ImmuneScore, and Tumor purity (**Figure 5A**). In specific breast cancer, POGLUT2 was positively correlated with StromalScore, ESTIMATEScore, and ImmuneScore and negatively correlated with Tumor purity (**Figure 5B**). The role of POGLUT2 on immune infiltration was also figured using the data from the TIMER2 dataset (**Figure 5C**). The results indicated that POGLUT2 was positively correlated with cancer-associated fibroblasts, macrophages, monocytes, and neutrophils and negatively correlated with B cells, T cells, and NK cells in most of the cancers. In specific breast cancer using the data from CIBERSORT, POGLUT2 was found positively correlated with monocyte, macrophage, and infiltration scores (**Figure 5D**) and negatively correlated with B cells, CD8 T cells, and conventional T cells. In a specific BRCA setting, POGLUT2 was found positively correlated with iTreg, macrophage, DC, and Th2 cells and negatively correlated with neutrophils (**Figure 5E**).

### 3.6 The association between POGLUT2 and immune-related genes

**Figure 6:**
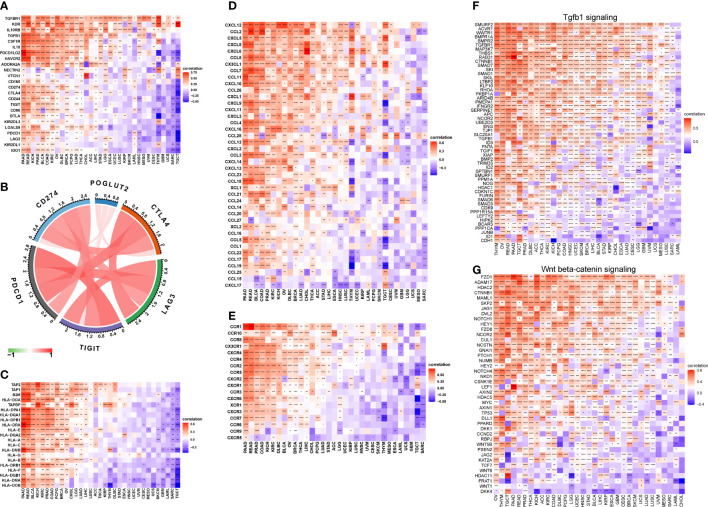
The association between POGLUT2 and immune-related genes. **(A)** The association between POGLUT2 and immune inhibitory genes in pan-cancer. **(B)** The association between POGLUT2 and immune checkpoint genes in breast cancers. **(C)** The association between POGLUT2 and MHC genes. **(D)** The association between POGLUT2 and chemokines. **(E)** The association between POGLUT2 and chemokine receptors. **(F)** The association between POGLUT2 and immune inhibitory genes in pan-cancer in specific signaling pathways. Left, TGF-beta1 signaling. Right, Wnt-beta-catenin signaling. **(G)** the top 3 POGLUT2 positively correlated genes were assessed.

On immune inhibitory genes, the top 3 POGLUT2 positively correlated genes were TGFBR1, KDR, and IL10RB, whereas the top 3 negatively correlated genes were IDO1, KIR2DL1, and LAG3 (**Figure 6A**). On immune checkpoint genes in breast cancers, POGLUT2 was positively correlated with CTLA4, CD274, TIGIT, LAG3, and PDCD1 (**Figure 6B**). On MHC genes, the top 3 POGLUT2 positively correlated genes were TAP2, TAP1, and B2M, whereas the top 3 negatively correlated genes were HLA-DOB, HLA-DMA, and HLA-DQB1 (**Figure 6C**). On chemokines, the top 3 POGLUT2 positively correlated genes were CXCL12, CCL2, and CXCL5, whereas the top 3 negatively correlated genes were CXCL17, CCL-15, and CCL-25 (**Figure 6D**). On chemokine receptors, the top 3 POGLUT2 positively correlated genes were CCR1, CCR10, and CCR8, whereas the top 3 negatively correlated genes were CXCR5, CCR9, and CCR6 (**Figure 6E**). Moreover, the association between POGLUT2 and immune inhibitory genes in pan-cancer in specific TGF-beta1 and Wnt-beta-catenin signaling pathways was also illustrated. In TGF-beta1 pathways, the top 3 POGLUT2 positively correlated genes were SMURF2, ACVR1, and WWTR1, whereas the top 3 negatively correlated genes were CDH1, ID1, and JUNB (**Figure 6F**). In Wnt-beta-catenin pathways, the top 3 POGLUT2 positively correlated genes were FZD1, ADAM17, and HDAC2, whereas the top 3 negatively correlated genes were DKK4, WNT1, and FRAT1 (**Figure 6G**).

### 3.7 POGLUT2 correlated drug resistance analysis

**Figure 7:**
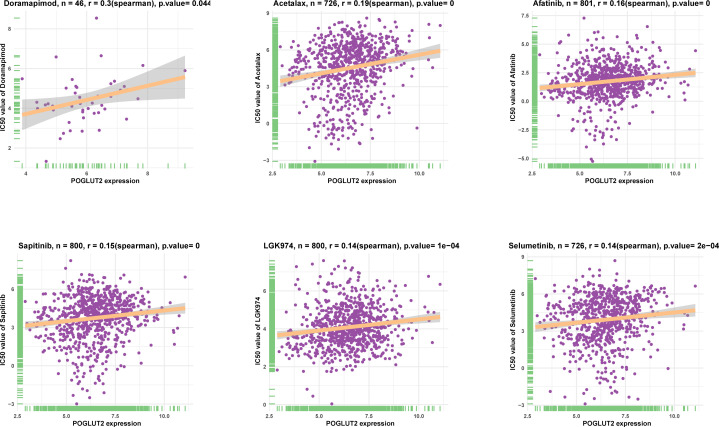
The top 6 POGLUT2 positively correlated drugs from GDSC2 database. The association between POGLUT2 and IC50 of 198 drugs was calculated through Spearman’s correlation.

The association between POGLUT2 and IC50 of 198 drugs was calculated through Spearman’s correlation, and the top 6 POGLUT2 positively correlated drugs from the GDSC2 database (**Figure 7**) were doramapimod, acetalax, afatinib, sapitinib, LGK974, and selumetinib.

### 3.8 Expression evaluation of POGLUT2 in breast cancer cells and tissues

**Figure 8:**
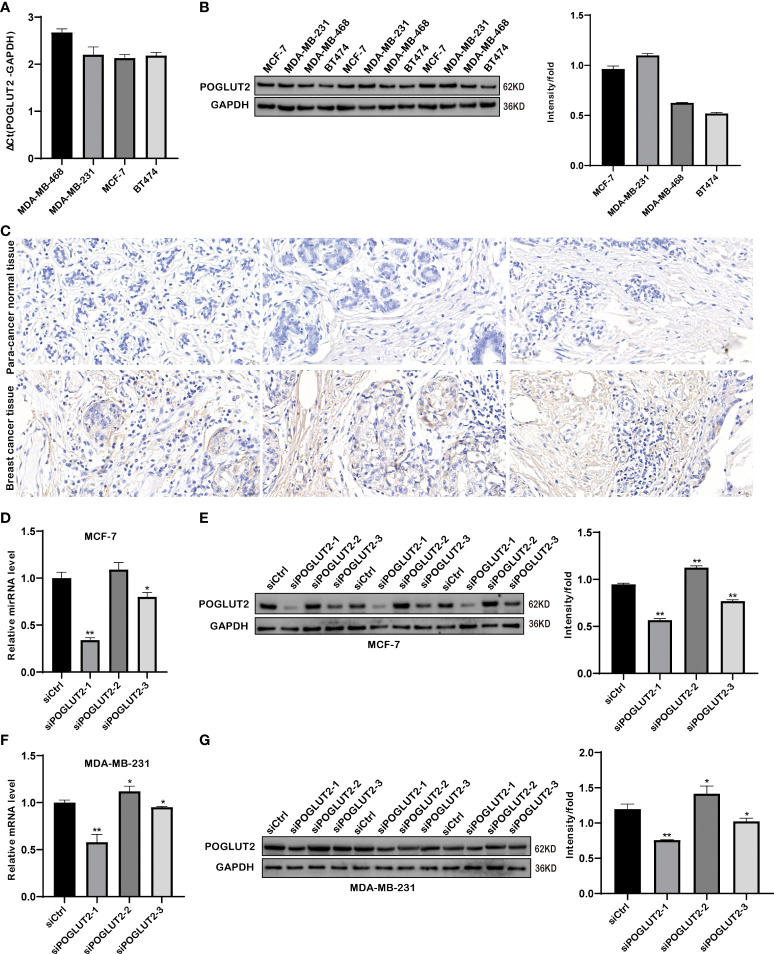
Gene and protein expression evaluation of POGLUT2 in breast cancer cells and tissues. **(A)** Gene expression of POGLUT2 was determined in four breast cancer cell lines, including MDA-MB-468, MDA-MB-231, MCF-7, and BT474, and highest expression was found in MCF-7 cells. **(B)** Protein expression of POGLUT2 was determined in four breast cancer cell lines, including MDA-MB-468, MDA-MB-231, MCF-7, and BT474, and highest expression was found in MDA-MB-231 cells. **(C)** Immunohistochemistry analysis of POGLUT2 in breast cancer tissues compared to control tissues. **(D)** Significant POGLUT2 expression downregulation was found in MCF-7 cells after siRNA transfection. **(E)** Significantly POGLUT2 expression downregulation was found in MDA-MB-231 cells after siRNA transfection. **(F)** Significantly POGLUT2 protein level downregulation was found in MCF-7 cells after siRNA transfection. **(G)** Significantly POGLUT2 protein level downregulation was found in MDA-MB-231 cells after siRNA transfection. *p < 0.05; **p < 0.01.

Expression of POGLUT2 was determined in four breast cancer cell lines, including MDA-MB-468, MDA-MB-231, MCF-7, and BT474, and the highest gene and protein expression were respectively found in MCF-7 and MDA-MB-231 cells (**Figures 8A, B**). Meanwhile, immunohistochemistry analysis of POGLUT2 in breast cancer tissues revealed increased levels of POGLUT2 compared to control tissues (**Figure 8C**). Moreover, we also established the POGLUT2 knockdown MCF-7 and MDA-MB-231 cells, and significantly decreased levels of gene and protein expression of POGLUT2 were found in these cells after siRNA transfection (**Figures 8D–G**).

### 3.9 POGLUT2 regulates the cell proliferation, apoptosis, cell cycle, clone formation, migration, and invasion in breast cancer cells *in vitro*

**Figure 9:**
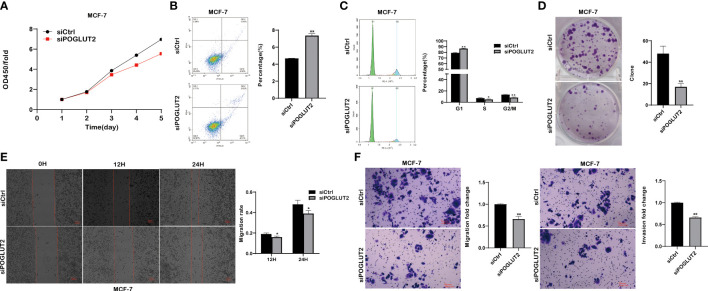
POGLUT2 regulates cell proliferation, apoptosis, cell cycle, clone formation, migration, and invasion in breast cancer cell line MCF-7 *in vitro*. **(A)** Decreased cell proliferation was found in POGLUT2 knockdown MCF-7 cells. **(B)** Increased cell apoptosis was found in POGLUT2 knockdown MCF-7 cells. **(C)** Increased G1 phase and decreased S and G2 phase in POGLUT2 knockdown MCF-7 cells. **(D)** Decreased cell clone formation in POGLUT2 knockdown MCF-7 cells. **(E)** Decreased cell migration of POGLUT2 knockdown MCF-7 cells in 2-D wound healing assay. **(F)** Decreased cell migration and invasion of MCF-7 cells in 3-D transwell assay. *p < 0.05; **p < 0.01.

**Figure 10:**
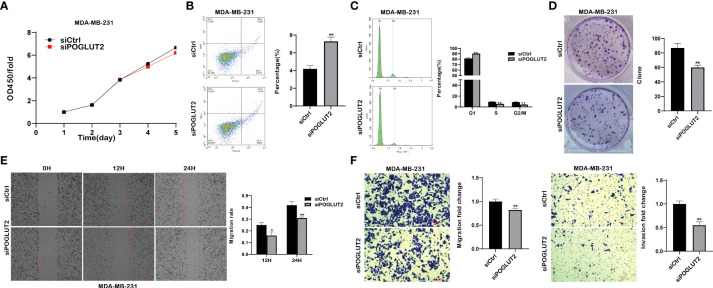
POGLUT2 regulates cell proliferation, apoptosis, cell cycle, clone formation, migration, and invasion in breast cancer cell line MDA-MB-231 *in vitro*. **(A)** Decreased cell proliferation was found in POGLUT2 knockdown MDA-MB-231. **(B)** Increased cell apoptosis was found in POGLUT2 knockdown MDA-MB-231. **(C)** Increased G1 phase and decreased S and G2 phase in POGLUT2 knockdown MDA-MB-231. **(D)** Decreased cell clone formation in POGLUT2 knockdown MDA-MB-231. **(E)** Decreased cell migration of POGLUT2 knockdown MDA-MB-231 in 2-D wound healing assay. **(F)** Decreased cell migration and invasion of MDA-MB-231 in 3-D transwell assay. *p < 0.05; **p < 0.01.

We then established POGLUT2 knockdown cell lines to examine their biological behavior; the results showed that decreased cell proliferation, increased cell apoptosis, increased G1 phase and decreased S and G2 phase, decreased cell clone formation, decreased cell migration in 2-D and 3-D systems, and decreased cell invasion in a 3-D system were found in POGLUT2 knockdown MCF-7 (**Figures 9A–F**) and MDA-MB-231 cells (**Figures 10A–F**).

### 3.10 POGLUT2 knockdown decreased the tumor growth *in vivo*

**Figure 11:**
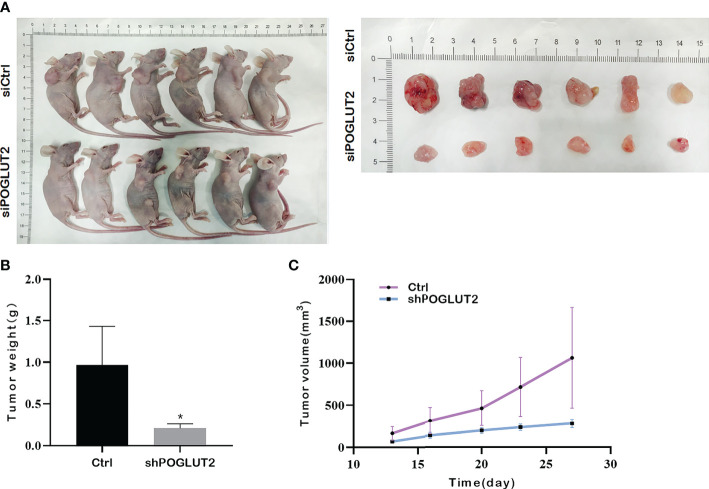
POGLUT2 knockdown decreased the tumor growth in a xenograft transplantation model. **(A)** Photographs of tumor-bearing nude mice and corresponding tumor tissues. **(B)** Line chart quantification indicated that deceased tumor volume was found in mice injected with POGLUT2 knockdown MCF-7 cells. **(C)** Bar graph quantification indicated decreased tumor weight in mice with POGLUT2 knockdown MCF-7 cells. *p < 0.05.

*In vivo* results indicated that POGLUT2 knockdown decreased the tumor growth as verified by tumor size, tumor volume, and tumor weight (**Figures 11A–C**).

### 3.11 Notch pathway is involved in the effects of POGLUT2 in breast cancer cells

**Figure 12:**
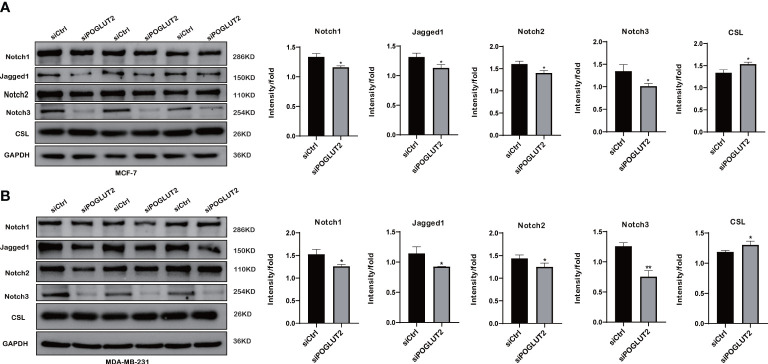
Notch pathway is involved in the effects of POGLUT2 in breast cancer cells. **(A)** Increased levels of CSL and decreased levels of Jagged1, Notch1, Notch2, and Notch3 were found in POGLUT2 knockdown MCF-7 cells. **(B)** Increased levels of CSL and decreased levels of Jagged1, Notch1, Notch2, and Notch3 were found in POGLUT2 knockdown MDA-MB-231 cells. *p < 0.05.

To further test the possible molecular mechanisms, we performed Western blotting to check the effects of POGLUT2 knockdown in two breast cancer cell lines. The results indicated that increased levels of CSL and decreased levels of Jagged1, Notch1, Notch2, and Notch3 were found in POGLUT2 knockdown MCF-7 and MDA-MB-231 cells (**Figures 12A, B**).

## 4 Discussion

Glycosylation is a kind of widespread and important protein post-translational modification process, which is catalyzed by glycosyltransferases (20). Specific glycosylation modifications are essential for protein activity, and they are presented in various proteins with clinical therapeutic purposes, such as antibodies and vaccines (21). The chemical enzymatic synthesis method based on glycosyltransferase is one of the effective ways to obtain uniform glycoproteins (22). Therefore, understanding the role and effects of glycosyltransferases in the biological process could help us to discover novel therapeutic targets regarding their biochemical properties.

According to previous reports, the Notch signaling pathway, as one of the most conserved cellular signaling pathways, plays a critical role in animal development and tissue homeostasis (23, 24). Recent visualization of Notch receptor structures in complex with Notch ligands and those of Notch-modifying glycosyltransferases has promoted the understanding of the significance of glycosylation in Notch signaling component regulation (25–27). In the present study, we evaluated the expression, gene mutation and amplification, methylation, and CNA of a novel *O*-glucosyltransferase POGLUT2 using TCGA, CCLE, and GTEx databases. Moreover, we also evaluated the role of POGLUT2 on survival and disease progression in pan-cancer based on TCGA data. Moreover, we also found that immune infiltration and tumor microenvironment evaluation using the ImmuneScore, ImmuCellAI, and TIMER databases and POGLUT2 correlated with drug resistance analysis using the GDSC2 database. Furthermore, POGLUT2 knockdown of breast cancer cells was established, followed by *in vitro* biological function assays and *in vivo* tumor growth study. Finally, we explored the mechanisms of POGLUT2 in breast cancer *via* a brief evaluation of its connection with Notch signaling.

Our results showed that increased levels of POGLUT2 were found in multiple types of cancer tissues and cell lines. Moreover, increased gene mutation and amplification, methylation, and CNA of POGLUT2 were found in several types of cancers. In recent studies by Williamson et al., POGLUT2 was found to modify not only a small fraction of Notch molecules with EGF repeats but also over half of fibrillin proteins with the EGF repeats (FBN1, FBN2, and LTBP1), which are the major components of extracellular matrix microfibrils and hold an important role in elastic tissue rational development, including the major organs (heart and lungs). Williamson et al. concluded that POGLUT2 together with *O*-glycosylated protein substrates is involved in the secretion of substrate proteins. In our cell knockdown experiment, we also found increased levels of CSL and decreased levels of Jagged1, Notch1, Notch2, and Notch3, which is consistent with Williamson’s study.

Here, we found that POGLUT2 was mainly expressed in stromal cells, which is verified by StromalScore, ESTIMATEScore, ImmuneScore, and Tumor purity, and POGLUT2 was positively correlated with cancer-associated fibroblasts, macrophages, monocytes, and neutrophils in the tumor microenvironment. These results indicated that POGLUT2 could regulate the immune status in the tumor microenvironment, thereby facilitating the development and progression of cancer. However, further studies are still required for obtaining detailed information about POGLUT2.

To confirm the specific function of POGLUT2 in human cancer, *in vitro* and *in vivo* validations were carried out. The results showed that POGLUT2 knockdown could delay the tumor growth and progression of breast cancer. Notch signaling components were related to the function of POGLUT2. According to the previous studies about POGLUT1, a member from the same family of POGLUT2, elevated POGLUT1 expression was found in hematopoietic malignancies, including primary acute myelogenous leukemia and T-ALL (28). Moreover, POGLUT1 amplification and overexpression were found in NSCLC (29). RNAi-mediated POGLUT1 knockdown in NSCLC cell lines A549 could result in a significant reduction in the expression of HEY1 and HES2, both of which are Notch downstream target genes that inhibit cell proliferation, migration, and survival. Furthermore, POGLUT1 was confirmed as a protein *O*-glucosyltransferase that transfers glucose and xylose to the EGF-like domains on Notch (30). In addition, based on the expression and clinical data, POGLUT1 was identified as a novel negative prognostic factor and could serve as a potential therapeutic target for NSCLC.

There are some limitations in our present study. The detailed cell identities related to POGLUT2 are not determined. Considering the prosperity of single-cell analysis technology (31), sorted POGLUT2-expressed cells might be collected for further analysis. Therefore, it is possible that novel mechanisms could be expected on POGLUT2. Additionally, the enhanced effect of POGLUT2 on breast cancer development has been confirmed in the present study. Due to the heterogeneity of gene expression and function, the specific role and mechanism of POGLUT2 need further investigations in various human cancers.

In summary, our results here demonstrated that increased levels of POGLUT2 in cancer could result in dysregulated immune cell infiltration and tumor microenvironment. Validated in breast cancer, the knockdown of POGLUT2 exerted a significant inhibitory effect on cell growth, apoptosis, and metastasis and suppressed tumor progression in rats by regulating Notch-related signaling.

## Data availability statement

The raw data supporting the conclusions of this article will be made available by the authors, without undue reservation.

## Ethics statement

The animal study was reviewed and approved by The Biomedical Ethics Committee of Gannan medical University.Written informed consent was obtained from the individual(s) for the publication of any potentially identifiable images or data included in this article.

## Author contributions

Conceived and designed the study: XX and QL. Performed the literature search and data extraction: GX. Analyzed the data: MX. Drafted the manuscript: XX and GX. All authors contributed to the article and approved the submitted version.

## Funding

This work was supported by the Key Projects of Nature Science Foundation of Jiangxi (grant no. 20192ABCL20005), the Nature Science Foundation of Jiangxi (grant no. 20181BAB205052), and The Technology Plan Fund of Jiangxi Province Department of Education Project (grant no. GJJ211534).

## Conflict of interest

The authors declare that the research was conducted in the absence of any commercial or financial relationships that could be construed as a potential conflict of interest.

## Publisher’s note

All claims expressed in this article are solely those of the authors and do not necessarily represent those of their affiliated organizations, or those of the publisher, the editors and the reviewers. Any product that may be evaluated in this article, or claim that may be made by its manufacturer, is not guaranteed or endorsed by the publisher.

## Glossary

**Table:**

ACC	adrenocortical carcinoma
BLCA	bladder urothelial carcinoma
BRCA	breast invasive carcinoma
CESC	cervical squamous cell carcinoma and endocervical adenocarcinoma
CHOL	cholangiocarcinoma
COAD	colon adenocarcinoma
DLBC	lymphoid neoplasm diffuse large B-cell lymphoma
ESCA	esophageal carcinoma
GBM	glioblastoma multiforme
HNSC	head and neck squamous cell carcinoma
KICH	kidney chromophobe
KIRC	kidney renal clear cell carcinoma
KIRP	kidney renal papillary cell carcinoma
LAML	acute myeloid leukemia
LGG	brain lower-grade glioma
LIHC	liver hepatocellular carcinoma
LUAD	lung adenocarcinoma
LUSC	lung squamous cell carcinoma
MESO	mesothelioma
OV	ovarian serous cystadenocarcinoma
PAAD	pancreatic adenocarcinoma
PCPG	pheochromocytoma and paraganglioma
PRAD	prostate adenocarcinoma
READ	rectal adenocarcinoma
SARC	sarcoma
STAD	stomach adenocarcinoma
TGCT	testicular germ cell tumors
THCA	thyroid carcinoma
THYM	thymoma
UCEC	uterine corpus endometrial carcinoma
UCS	uterine carcinosarcoma
UVM	uveal melanoma
